# Multi-jet propulsion organized by clonal development in a colonial siphonophore

**DOI:** 10.1038/ncomms9158

**Published:** 2015-09-01

**Authors:** John H. Costello, Sean P. Colin, Brad J. Gemmell, John O. Dabiri, Kelly R. Sutherland

**Affiliations:** 1 Eugene Bell Center, Marine Biological Laboratory, Woods Hole, Massachusetts 02543, USA; 2 Department of Biology, Providence College, Providence, Rhode Island 02918, USA; 3 Department of Marine Biology and Environmental Science, Roger Williams University, Bristol, Rhode Island 02809, USA; 4 Department of Integrative Biology, University of South Florida, Tampa, Florida 33620, USA; 5 School of Engineering, Stanford University, Stanford, California 94305, USA; 6 Oregon Institute of Marine Biology, University of Oregon, Eugene, Oregon 97403, USA

## Abstract

Physonect siphonophores are colonial cnidarians that are pervasive predators in many neritic and oceanic ecosystems. Physonects employ multiple, clonal medusan individuals, termed nectophores, to propel an aggregate colony. Here we show that developmental differences between clonal nectophores of the physonect *Nanomia bijuga* produce a division of labour in thrust and torque production that controls direction and magnitude of whole-colony swimming. Although smaller and less powerful, the position of young nectophores near the apex of the nectosome allows them to dominate torque production for turning, whereas older, larger and more powerful individuals near the base of the nectosome contribute predominantly to forward thrust production. The patterns we describe offer insight into the biomechanical success of an ecologically important and widespread colonial animal group, but, more broadly, provide basic physical understanding of a natural solution to multi-engine organization that may contribute to the expanding field of underwater-distributed propulsion vehicle design.

## Abstract (web summary)

Physonect siphonophores are highly mobile jellyfish with complex colonial organization. Here, Costello *et al*. show that division of labour among developmental stages controls the direction and propulsion of the colony, with older individuals providing thrust and younger individuals providing torque.

## Graphical abstract

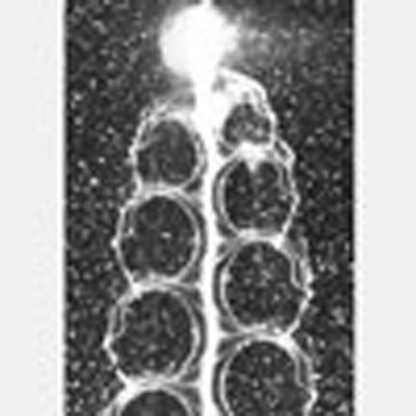

Although jet propulsion is common among animal swimmers^1^, the integration of multiple jets into a coordinated propulsive whole is rare within the animal kingdom. Directional control of a single jet typically relies on manipulation of the jet aperture to control orientation of thrust production during animal swimming^2^,^3^,^4^. Analogous directional aperture control during manoeuvring, termed thrust vectoring, is used for jet propulsion in human-engineered vehicles^5^,^6^. For both animals and vehicles, thrust vectoring permits substantial directional control of the propelled body. However, this same latitude of motion could prove counterproductive in a multi-jet array if the individual units do not act synergistically. Consequently, collaborative interactions among propulsive units are critical for colonial swimmers. In addition, the growing sophistication of distributed propulsion vehicles^7^,^8^ depends on increased understanding of propulsor coordination and opens the potential for biologically inspired solutions to contribute to human-engineered underwater vehicle designs.

Physonect siphonophores are a group of colonial cnidarians that possess the most complex colony-level organization of all animals^9^,^10^ and have evolved a highly sophisticated multi-jet propulsion system. Physonects employ an array of coordinated jets produced by specialized colony members (Fig. 1) to propel an aggregate colony through the water column. The jet-producing members of the physonect colony are termed nectophores and are genetically identical clones arranged within a coherent unit, termed the nectosome. The nectosome pulls feeding and reproductive colony members, arranged within a portion of the colony termed the siphosome, during whole-colony swimming. Physonect siphonophores comprise a highly successful marine planktonic taxon and most species are distributed over wide oceanic regions^11^,^12^.

One of the most commonly encountered physonect species, *Nanomia bijuga*, is a widely distributed species that is seasonally numerous and is known to play a dominant predatory role in planktonic communities^13^. A highly proficient swimmer^14^, *N. bijuga* rapidly alters course during complex turns and is capable of completely reversing the direction of whole-colony propulsion. These colonies may migrate hundreds of metres daily between deeper layers during daylight and near-surface during night hours^13^,^15^. Placed on a human size scale, a *N. bijuga* colony possessing slightly over a 2-cm-long nectosome that migrates 200 m daily, performs the equivalent of a 2-m-long human running a marathon daily while towing behind them a load equal or greater than their body mass. These daily vertical migrations of *N. bijuga* involve enough colonies to periodically dominate variations in oceanic acoustic deep-scattering layers^16^.

Here we show that developmental differences between nectophores of the physonect *N. bijuga* produce a division of labour in thrust and torque production by individuals within the nectosome. These patterns permit all members of the colony—from younger, smaller individuals to older, larger individuals—to make important contributions to the propulsion and manoeuvring traits that are critical for the success of *N. bijuga* in their natural environment. The integration of different individuals into a natural solution of multi-engine organization may contribute to the expanding field of underwater-distributed propulsion vehicle design.

## Results

### Nectophore developmental characteristics

Recorded video sequences of *N. bijuga* colonies and the fluid motions surrounding their bodies during colonial swimming demonstrated that, although nectophores form by budding and are genetically identical, their developmental pattern produces distinct age-dependent patterns within the nectosome. The most recently formed nectophores at the anterior end of the nectosome are comparatively small and their resting velar apertures are oriented more parallel to the nectosomal central axis than those of older nectophores (Fig. 2a–c). As a colony grows and an individual nectophore ages, attainment of maximum size is accompanied by a shift in velar orientation so that the oldest nectophores are characterized by resting velar apertures that are more divergent from the nectosomal axis orientation. These developmental patterns of velar orientation affect the directions of the propulsive jets produced by nectophores of differing age and position within the nectosome (Fig. 2d). Although *N. bijuga* can swim in either forward or reverse directions (Supplementary Movies 1 and 2 and Supplementary Fig. 1), forward swimming is their predominant swimming mode and, after an initial synchronous contraction by the entire nectosome, forward swimming typically involves asynchronous contractions (Supplementary Movie 3) by different members within the nectosome^14^. During forward swimming, the jets of the youngest, most anterior nectophores were directed significantly more normally to the nectosome axis than those of older nectophores, which were directed more parallelly to the nectosome axis (Fig. 2d and Supplementary Movie 4). The direction of jet flows relative to the nectosome axis was relatively steady during nectosac contraction for forward swimming (Supplementary Fig. 2) and did not significantly vary whether swimming synchronously or asynchronously (Fig. 2d), or while moving in a linear path or turning (Supplementary Fig. 3).

### Nectophore performance characteristics

**Figure 1:**
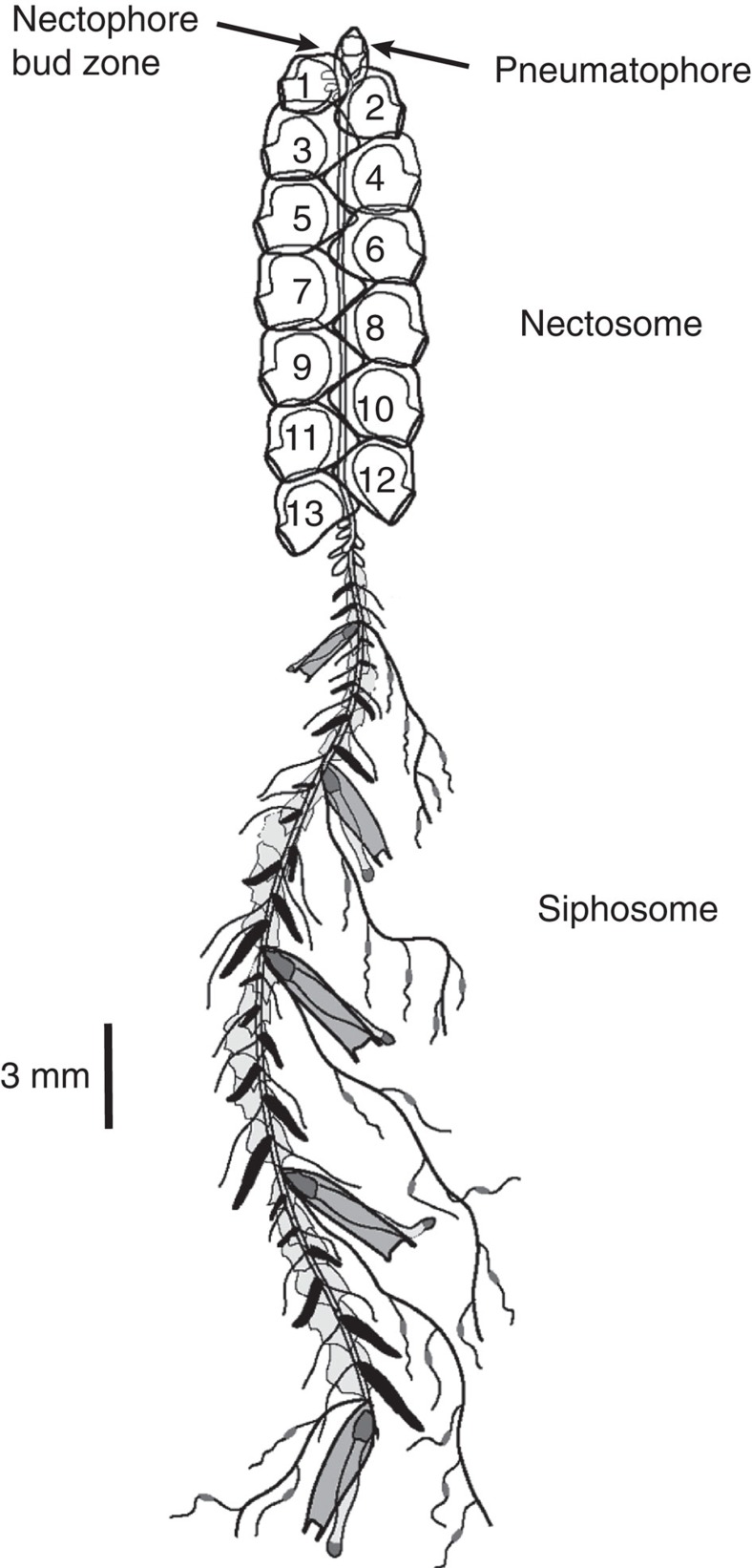
*N. bijuga* body structure. The propulsive component of the colony consists of the nectophores that bud sequentially (numbered in order of their origin) from a budding zone near the pneumatophore float at the anterior region of the nectosome. Nectophores lie in one plane bisected by the nectosomal axis. The siphosome includes the feeding and reproductive members of the siphonophore colony. (Image modified from ref. 12 licenced under CC BY 4.0).

**Figure 2:**
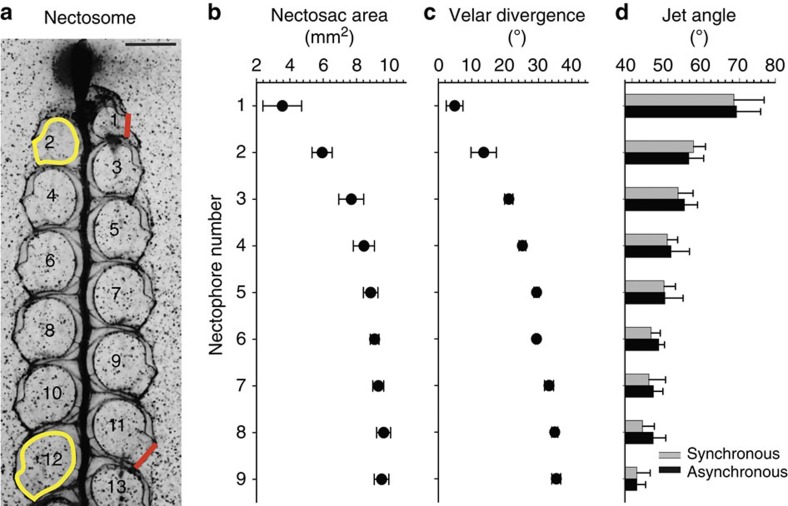
Developmental patterns of nectophore structure. Morphological characteristics of nectophore developmental stages (nectophores are numbered by their order of development) within a (**a**) resting nectosome, including (**b**) nectosac cross-sectional area (*n*=3 colonies for **b**,**c**), (**c**) angular divergence of the velar opening from that of the nectosome central axis (0° represents parallel, whereas 90° represents perpendicular with jet directed towards the posterior of the colony) and (**d**) ejected fluid jet angle relative to the nectosome central axis during forward swimming. Nectosac cross-sectional area (yellow lines in nectophores 2 and 12) and resting velar orientation (red lines for nectophores 1 and 11) illustrate the morphological traits of representative nectophores in **a**. Scale bar, 3 mm in length. All error bars represent one s.d. from the mean value and are encompassed within the data mean where bars are not visible. Jet angles (**d**) represent forward swimming (*n*=6 colonies and asynchronous *n*=5 colonies). Jet angles significantly declined from the apical nectophore to older nectophores (factorial ANOVA, *P*<0.01) and that pattern was not significantly different between synchronous or asynchronous swimming modes (factorial ANOVA, *P*=0.37) nor were there significant interactions between different nectophores with swimming modes (*P*=0.99).

**Figure 3:**
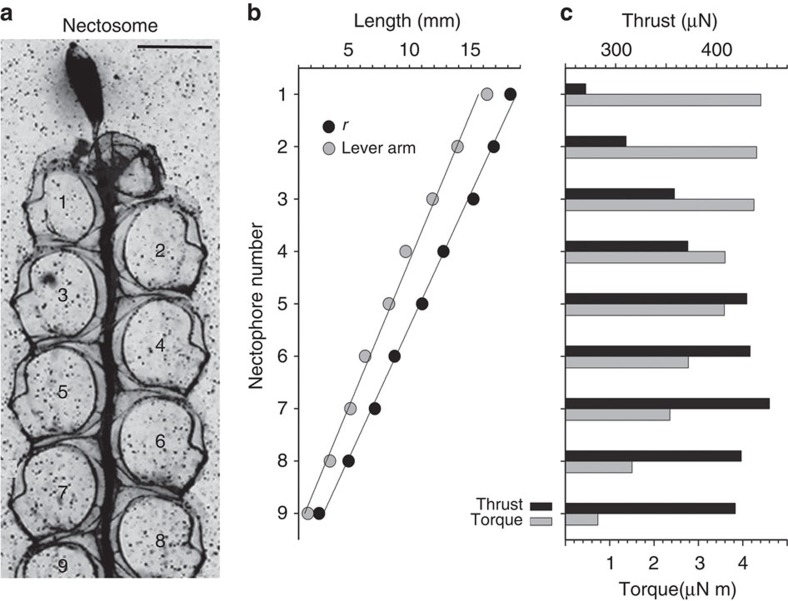
Performance consequences of nectophore developmental patterns. Impact of developmental stage and spatial position for individual nectophores of a (**a**) nine-member *N. bijuga* nectosome during forward swimming. Scale bar, 3 mm in length. (**b**) The youngest nectophores are located most distantly from the nectosome–siphosome border (distance *r*) and possess the longest lever arms within the nectosome. Nectophore developmental stage (*x*) is linearly related to *r* (*r*=−1.96*x*+20.65, *r*^2^=0.99, *P*<0.001) and lever arm (lever arm=−1.77*x*+17.38, *r*^2^=0.99, *P*<0.001) distances. The slopes of those two regressions are significantly different (analysis of covariance, df=2, *F*=1,480, *P*<0.001) because the lever arm distance integrates the angle of force application as well as the distance *r*. (**c**) The longer lever arms of the youngest, smallest nectophores enable them to produce comparatively high amounts of torque that affect the nectosome orientation during swimming. In contrast, the larger, more mature nectophores contribute relatively low torque levels but greater forward-directed thrust during whole-colony swimming.

**Figure 4:**
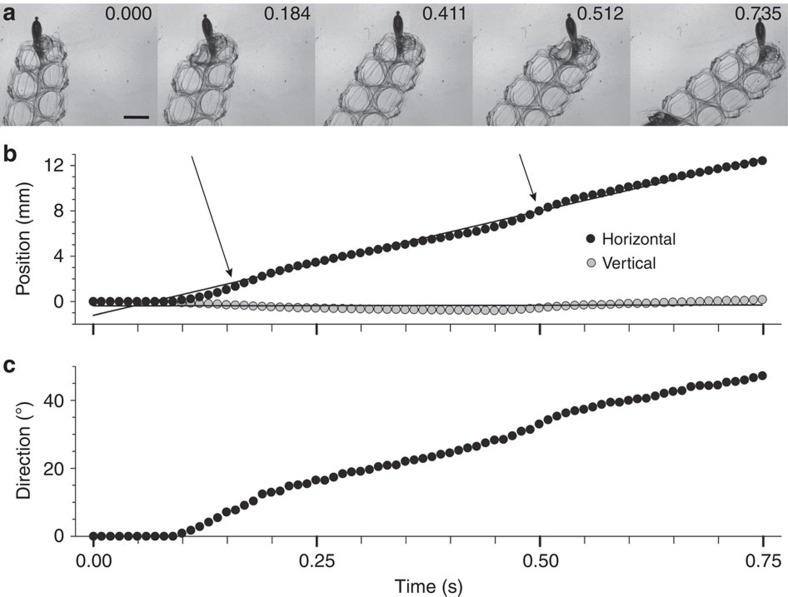
Effect of torque application by a single anterior nectophore on whole-colony direction during swimming. (**a**) Change in siphonophore colony orientation over two successive contractions of an anterior nectophore in a nine-member nectosome. The frame of reference was constant for all panels and contraction occurred in alternate panels (0.184, 0.512 s). Scale bar, 3 mm in length. (**b**) Change in position of the nectosome. Note that contraction by the anterior nectophore produced negligible vertical movement (linear regression, slope not significantly different than 0, *P*=0.48) while generating significant horizontal movement (*y*=18.4*x*−1.2, *r*^2^=0.99, *P*<0.001) and (**c**) altering the direction (∼50°) of the colony central nectosomal axis.

**Figure 5:**
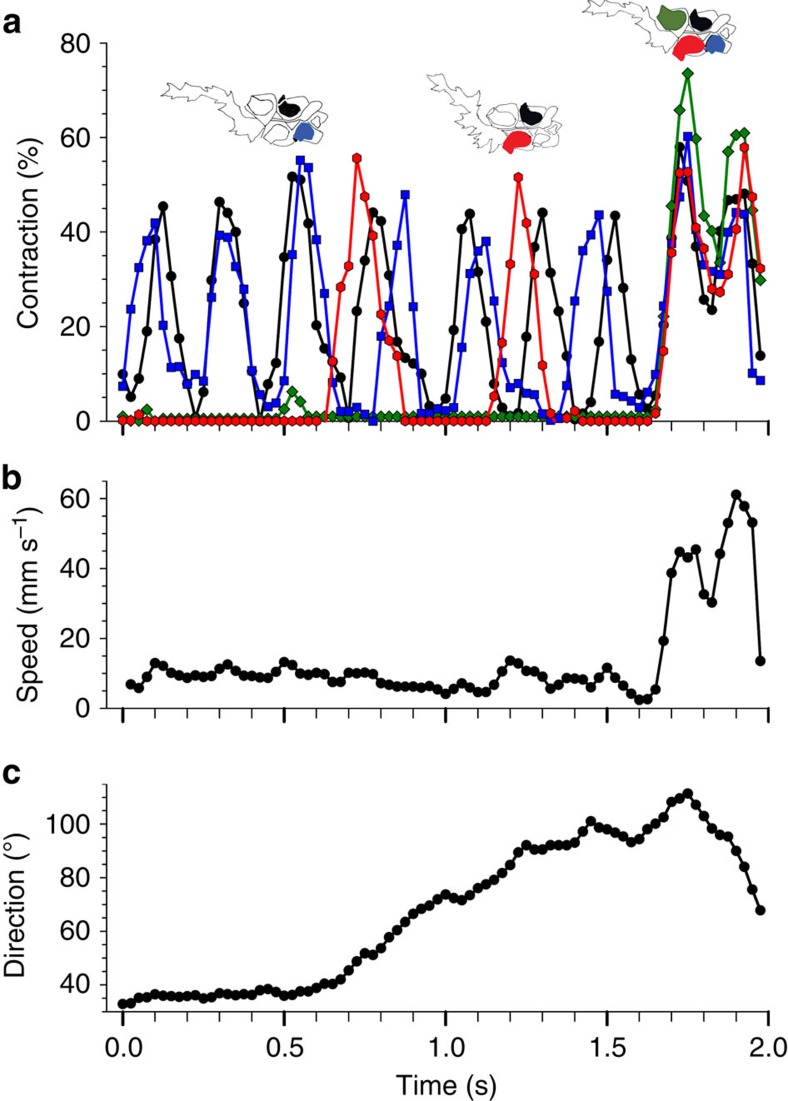
Cycles of nectophore contraction for a single four-member nectosome during forward asynchronous (0–1.6 s) and reverse synchronous (1.6–2.0 s) swimming. (**a**) The per cent contraction was measured as the change in nectosac (the fluid-filled interior of a resting nectophore) cross-sectional area relative to the relaxed state. The different nectophores are designated by colour as in the icons above the contraction data. (**b**) Whole-colony speed. (**c**) Angular direction of the colony. Note that during the initial observation period (0–0.6 s) the colony swam with relatively consistent direction and speed. The two youngest, anterior-most nectophores contracted nearly simultaneously during that period and their balanced contractions maintained stable directionality. Alterations in this pattern, including the additional contraction of the larger, older nectophores altered the angular path of the colony. Contraction extent varied during the period and was most extensive during the synchronous, reverse swimming that is associated with escape swimming.

Whole-colony directions and velocities during forward swimming were determined by the identities and extents of nectophore contractions. The spatial position of the youngest nectophores (Fig. 2a), coupled with their more normal jet trajectories (Fig. 2d), resulted in comparatively long lever arms (Fig. 3b and Supplementary Fig. 4) for jet force application to the nectosome. Consequently, despite their small size, the youngest, anterior-most nectophores exerted the greatest torque on the nectosome (Fig. 3c) and dominated whole-colony turning (Fig. 4 and Supplementary Movie 5). As a result, sequential contraction by a single anterior nectophore, while all other nectophores remain inactive, can result in turning of the entire nectosome and, subsequently, the whole siphonophore. However, the weaker jet production of younger, smaller nectophores resulted in lower contributions to forward thrust (calculations described in Methods and Supplementary Fig. 4) of the entire siphonophore colony relative to older, larger nectophores. In contrast, the older and larger nectophores closer to the siphosome budding zone produced larger, more powerful, backwardly directed jets with a lower angular trajectory relative to the nectosome axis. The relative spatial position and the jet trajectories of the older nectophores resulted in short lever arms that contributed comparatively little torque for whole-colony turning (Fig. 3b,c). The net direction and velocity of colonial swimming integrated the contributions of different nectophores to whole-colony propulsion. Frequent pulsing by the youngest nectophores on either side of the nectosome apex acted antagonistically to maintain a consistent heading during normal forward swimming (Fig. 5). Rotational motion of the nectosome allowed the planar nectophore array (Fig. 1) to spiral during swimming. Alterations in swimming speeds depended on both the number of high-thrust producing, older nectophores activated as well as the degree to which they contracted. We observed combinations of nectophore actuation that included: sole contraction by individual nectophores (for example, Fig. 4), paired antagonistic contractions by anterior nectophores on either side of the nectosome (Fig. 5), synchronous contraction of only one side of a nectosome and synchronous contraction by all nectosome members for either forward or reverse swimming (for example, Fig. 5). The range of nectophore-activation patterns as well as the potential extent of contractions by individual nectophores enabled a wide spectrum of swimming manoeuvres by *N. bijuga*.

## Discussion

Comparative nectophore performance demonstrated a persistent theme—all developmental stages contributed important components to colonial movement. The division of labour among nectophores assured that even the smallest individuals with lowest thrust production performed a critical role, turning, while the largest individuals with the highest thrust production contributed most to attain maximum forward or backward velocities. Both traits are important for the long diurnal vertical migrations undertaken by field populations of *N. bijuga.* Division of labour among these clonal units depended on an ontogenetic programme that positioned young individuals at the nectosome apex. Subsequent allometric development altered both morphology and function as the nectophore aged. Consequently, young nectophores were not merely small adults, but, instead, developed at a location and with jet orientations that produced the high torque required for colonial turning. Although the lever arms of apical nectophores were primarily determined by their spatial position (Fig. 3b), the angle of force application by nectophore jets (the jet angles—Fig. 2d and Supplementary Fig. 2) also substantially affected their lever arms and, consequently, torque production by apical nectophores. For example, if the apical nectophore of the colony described in Fig. 3 (nectophore 1, jet angle 68°) were to alter its jet angle to that of the oldest, most basal nectophore (nectophore 9—jet angle 41°), the apical nectophore would experience an ∼25% decrease in torque production during contraction. Similarly, shifting mature nectophore jet angles to orientations more normal to the nectosomal axis would do little for nectosomal turning because of the close proximity of the mature nectophores to the rotational axis (and hence, low *r* values, Fig. 3b), but would diminish their contribution to forward thrust during forward swimming. The developmental programme also effectively employed a size-dependent trait common to medusae—pulse frequencies of smaller individuals exceed those of larger individuals within a species^17^,^18^,^19^. This size-dependent trait favours high-frequency contractions by the apical nectophores (Fig. 5a) and allows the youngest, smallest nectophores to function as bow thrusters that continually adjust orientation of the swimming colony.

The mechanics of directional control by *N. bijuga* show some important differences from contemporary human-engineered multi-jet systems. The latter depend on thrust vectoring. In contrast, *N. bijuga* appears to vary jet direction very little during forward swimming (Supplementary Fig. 2). *N. bijuga* is clearly capable of altering jet orientation within a pulsation cycle during either forward or reverse (Supplementary Fig. 5) swimming, but instead depends on stereotypic jet directionality during swimming. Overall, except during the alteration between forward and reverse swimming, thrust vectoring does not appear to be an important contributor to directional control during forward swimming by *N. bijuga*. Instead, *N. bijuga* appears to have harnessed developmental differences of propulsor position, morphology and performance in a coordinated array to control directional orientation.

One major evolutionary advantage of colonial siphonophore organization is the ability to chain multiple, small, individuals together to produce larger, aggregate colonies. The combination of multiple jet-producing nectophores into specialized colonial units has allowed siphonophores to exceed body dimensions that constrain individual medusae^17^,^20^,^21^. Multi-engine organization also permits a degree of redundancy among units that minimizes functional degradation should individual units suffer damage. However, these advantages of colonial organization are accompanied by the challenge of organizing effective cooperation among colony members. The developmental programme evolved by *N. bijuga* assures that all nectophore stages, from youngest to oldest, contribute in different yet important ways to whole-colony function.

## Methods

### Animal collection

*N. bijuga* colonies were collected from the floating docks at Friday Harbor Laboratories, San Juan Island, WA, USA, during June 2013 and 2014. Colonies were hand-collected with beakers most successfully at night using an underwater light to illuminate surface waters. Several colonies were also collected during daylight hours, although successful daylight collection was infrequent. Colony nectosomes ranged in size from 4 to 12 nectophores (<2.0 cm total nectosome length). Collected colonies were transported to the laboratory where they were maintained within sea tables with circulating seawater of approximately the same temperature (10–12 °C) as the collection site.

### Imaging

Images of *N. bijuga* were collected using either of two techniques—bright-field collimated light^22^ or dark-field laser sheet illumination^23^,^24^. All images were taken at 1,000 frames per second using a Photron Fastcam SA2 video camera. The collimated light technique provided adequate illumination to permit shutter speeds varying between 1/40,000 and 1/240,000 s and was useful for high resolution of nectophore-swimming kinematics. Laser sheet illumination^25^ used shutter speeds of 1/1,000 s to highlight particle movements relative to *N. bijuga* colony motions. Individual colonies were placed into glass filming vessels in filtered seawater seeded with 10-μm-hollow glass beads. The <1-mm-thick laser light sheet (535 nm wavelength) was oriented perpendicular to the camera view for two-dimensional (2D) quantification of particle velocity and direction data. Filming vessels ranged in size, depending on colony dimensions, from 8 × 2 × 4 to 15 × 4 × 12 (height × depth × width in cm).

### Image analysis

Measurements of body morphology, kinematics and particle motion were extracted from images using the ImageJ software (NIH). Jet velocities were measured by tracking individual particles at intervals throughout the nectophore contraction cycle. Nectophores and their velar apertures form a planar, linear array (Fig. 1) along the nectosome^14^,^15^, and particle tracks were collected from image sequences in which the planar nectophore arrays were normal to the camera. The velar apertures of the nectophores on either side of the nectosome central axis were in clear focus and perpendicular to the viewing field within particle-tracking sequences. Nectophore contraction duration differed depending on nectophore developmental stage (youngest nectophores had shorter contraction durations), and sample intervals varied accordingly (intervals of either 0.01 or 0.015 s). Replicate particles (*n*=5) were tracked for each sample interval, unless reliable particle tracks were not available and the total number of tracked particles (average=32, s.d.=11.1) per nectophore during a contraction cycle were used to determine an average flow velocity during nectophore contraction for thrust estimation. Jet angle measurements were made using replicate particle tracks (average=5 tracks per nectophore contraction, s.d.=1.9) relative to the nectophore central axis (Supplementary Fig. 4).

### Jet force and torque estimation

Jet thrust force (*F*) was calculated as


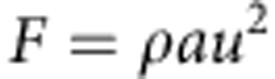


on the basis of the density of seawater (*ρ*), the area of the velar opening (*a*), estimated by assuming a circular opening with a diameter measured directly from contracting nectophore images, and jet velocity (*u*) measured by exit velocities of particles in an individual nectophore's jet.

Torque of individual nectophores assumed that the axis of rotation of the nectosome occurred at the juncture of the nectosome base with the remainder of the siphosome (Fig. 1). During swimming, this nectosome–siphosome junction is the location at which alterations in nectosome direction are transmitted to the trailing siphosome. The distance (*r*) from the axis of rotation to the line of jet force application at an individual nectophore was measured directly (Supplementary Fig. 4) and the lever arm (*L*) for force application calculated as:


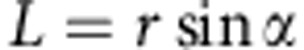


where *α* is the angle of jet force application to the nectosomal axis at a nectophore. Torque force application at a nectophore (*T*) was then estimated as


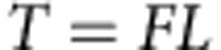


## Additional information

**How to cite this article:** Costello, J. H. *et al*. Multi-jet propulsion organized by clonal development in a colonial siphonophore. *Nat. Commun.* 6:8158 doi: 10.1038/ncomms9158 (2015).

## Supplementary Material

Supplementary Information Supplementary Figures 1-5.

Supplementary Movie 1 Forward, synchronous swimming by the siphonophore Nanomia bijuga.

Supplementary Movie 2 Reverse, synchronous swimming by the siphonophore Nanomia bijuga.

Supplementary Movie 3 Forward, asynchronous swimming by the siphonophore Nanomia bijuga.

Supplementary Movie 4 Laser light sheet illumination of particle tracks generated during forward, synchronous swimming by the siphonophore Nanomia bijuga.

Supplementary Movie 5 Whole-nectosome turning generated by sequential contraction of a single apical nectophore of the siphonophore Nanomia bijuga.
