# Supplementary material for: Multi-jet propulsion organized by clonal development in a colonial siphonophore
Source: Nat Commun. 2015 Sep 1;6:8158. doi: 10.1038/ncomms9158 (PMC4569723; doi:10.1038/ncomms9158)
Supplement: Supplementary Information — Supplementary Figures 1-5. [file ncomms9158-s1.pdf]

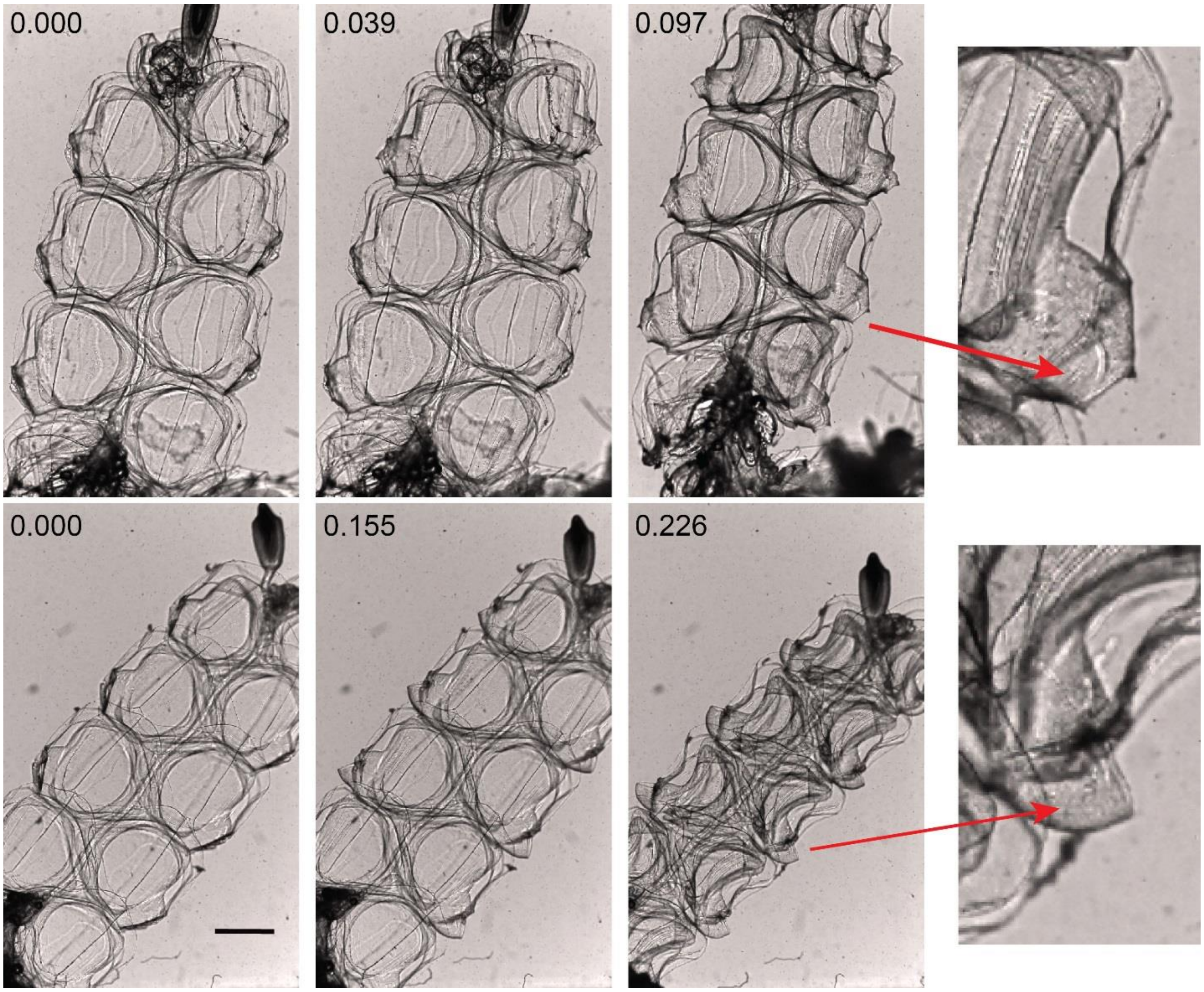

Supplementary Figure 1. Velum alterations during forward (top) and reverse (bottom) synchronous swimming events for two different *Nanomia bijuga* colonies. The time course (seconds) of nectophore contraction and velum eversion is shown in upper left of each panel, the spatial scale in bottom left panel. Arrows indicate enlarged image of velum position during nectophore contraction. Scale bar represents 2 mm in length. Jet directions resulting from nectophore contractions are essentially bimodal[14] and controlled by velar movements either forwards or backwards relative to the pneumatophore and nectosomal axis. These two modes of velar orientation result in either forward (top) or reverse (bottom) swimming by the colony.

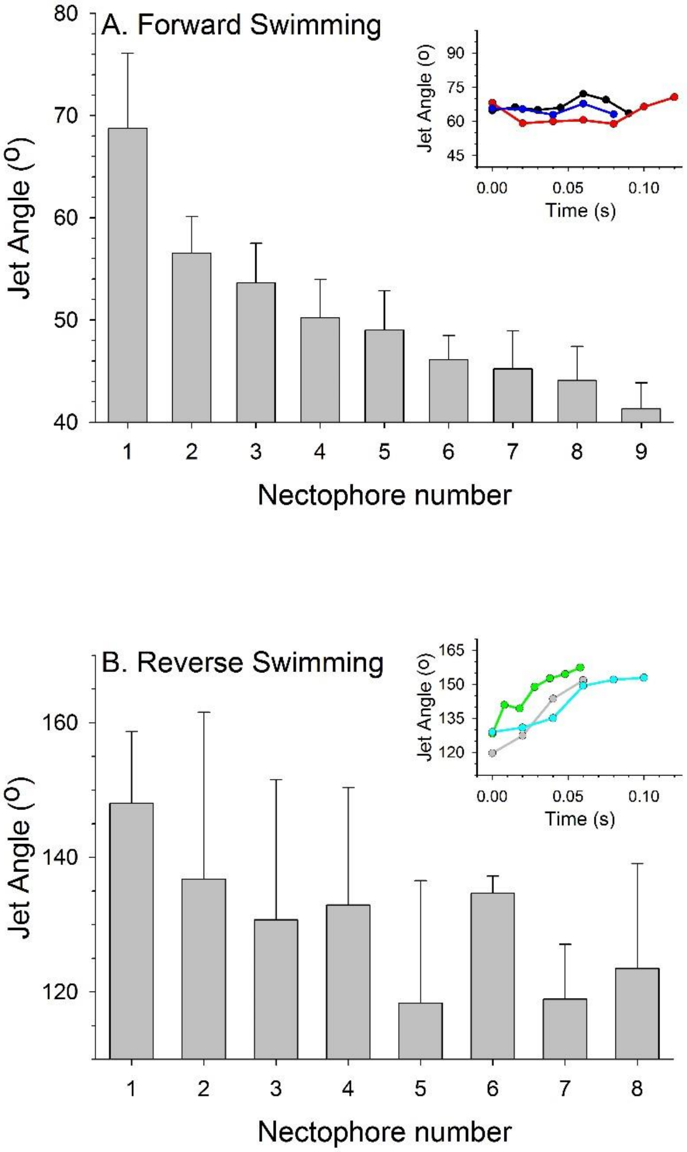

Supplementary Figure 2. Jet angles relative to nectophore central axis during synchronous swimming in the (A) forward (n = 11 colonies) and (B) reverse (n = 6 colonies) directions. Error bars represent 1 s.d. The inserted figures represent the time course of jet direction relative to the nectosomal axis during a nectophore contraction for the youngest active nectophore of three different colonies (each colony represented by a different color). Jet angles did not vary significantly over the time of the contraction cycle during forward swimming (linear regression, $p > 0.45$ for each of the nectophores) but increased significantly during contraction during reverse synchronous swimming (linear regression, $p < 0.02$ for each of the nectophores). Forward swimming is the dominant mode of propulsion by *N. bijuga* while reverse swimming is most frequently a transient escape response[14].

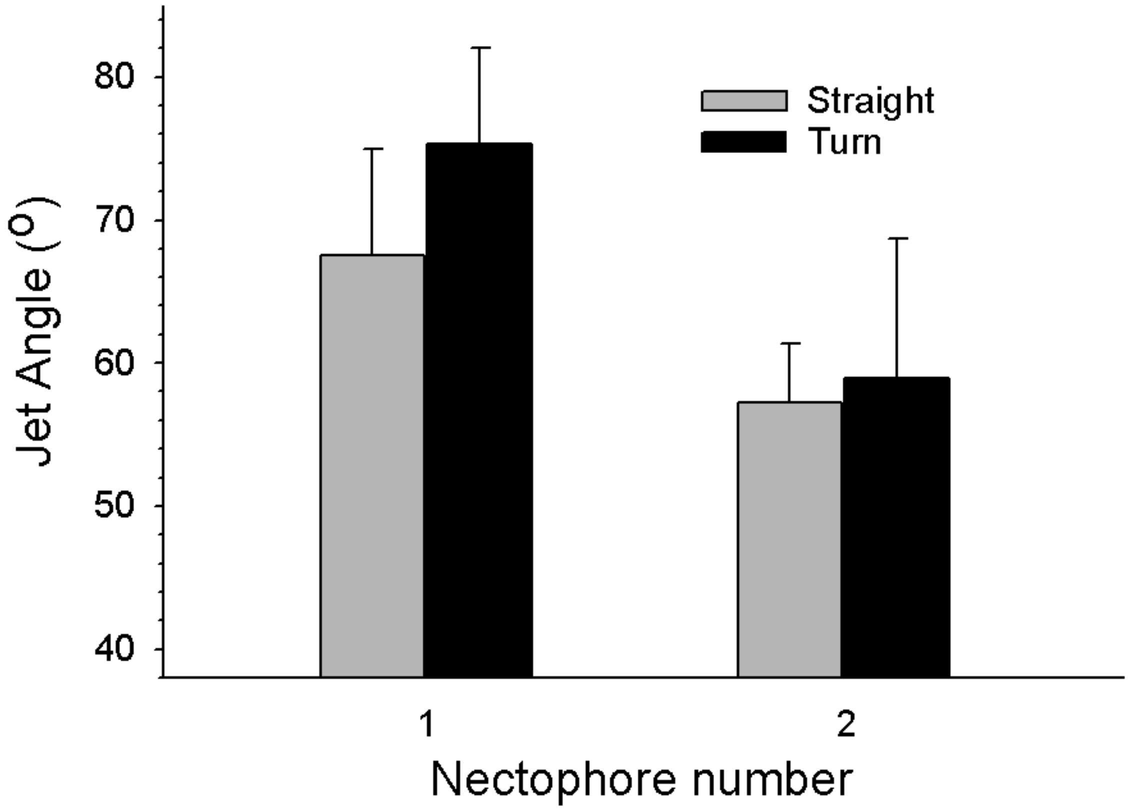

Supplementary Figure 3. Comparison of nectosome jet angles during straight swimming or turning by *Nanomia bijuga* colonies. Error bars represent 1 s.d. Only the apical nectophores were compared because although straight swimming involved multiple nectophores, turning often involved only a single apical nectophore. Although jet angles of nectophores1 and 2 were significantly different, jet angles for either nectophore did not significantly differ during straight swimming (n = 8 colonies for nectophore 1, 7 colonies for nectophore 2), or turning (n = 6 colonies for nectophore 1, 5 colonies for nectophore 2) (factorial ANOVA, p = 0.11), nor were there significant interactions between nectophore number and the direction of swimming (p = 0.29). Overall, these comparisons indicate that jet angles during forward swimming did not significantly vary for nectophores and were independent of alterations in swimming mode or direction.

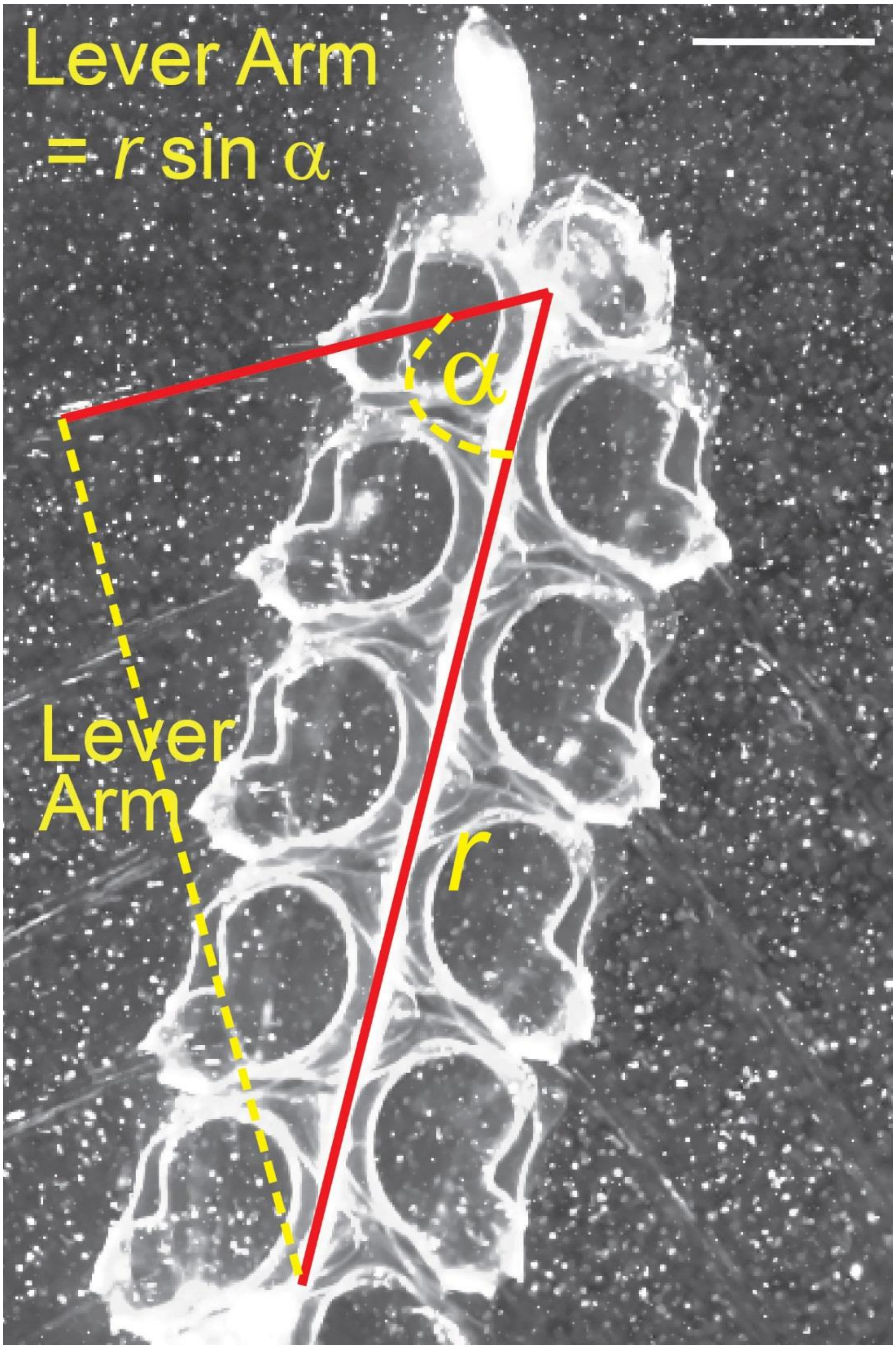

Supplementary Figure 4. Calculation of lever arm distance (*L*) used in torque estimation of the nectophores. The lever arm (yellow dashed line) for the top left nectophore is shown based on the distance (*r*) from the top of the siphosome budding region to the intersection of the angle (α) between the nectophore jet and the central nectosome axis. Scale bar (white solid line) represents 3 mm in length.

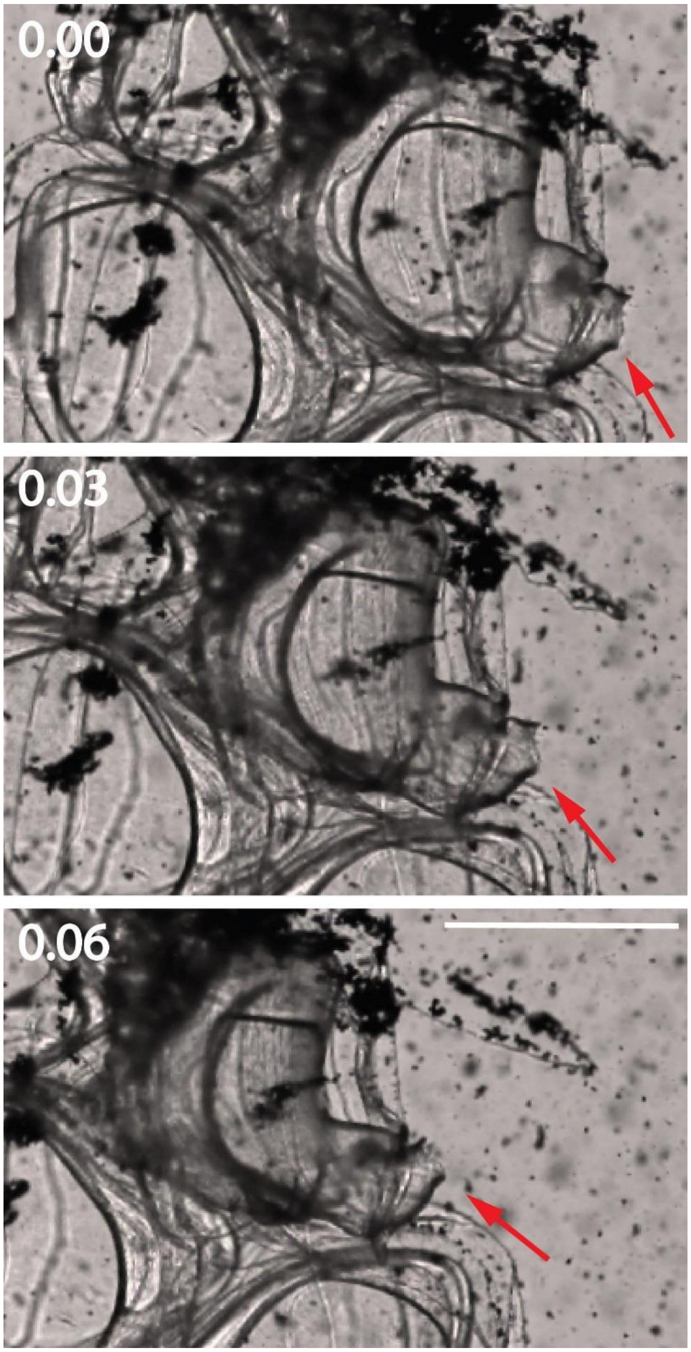

Supplementary Figure 5. Thrust vectoring by an apical nectophore of the siphonophore *Nanomia bijuga*. The nectophore velar aperture altered orientation and redirected the jet flow within a contraction during nectosomal turning controlled solely by the pictured contracting nectophore. Red arrows indicate the nectophore velum opening at sequential contraction stages. Time code refers to relative time (s) within the contraction cycle. Scale bar (white solid line at 0.06 s) represents 3 mm in length.
